# Validation of Pooled Whole-Genome Re-Sequencing in *Arabidopsis lyrata*

**DOI:** 10.1371/journal.pone.0140462

**Published:** 2015-10-13

**Authors:** Marco Fracassetti, Philippa C. Griffin, Yvonne Willi

**Affiliations:** 1 Institute of Biology, Evolutionary Botany, University of Neuchâtel, Neuchâtel, Switzerland; 2 School of BioSciences, University of Melbourne, Parkville, Victoria, Australia; Oklahoma State University, UNITED STATES

## Abstract

Sequencing pooled DNA of multiple individuals from a population instead of sequencing individuals separately has become popular due to its cost-effectiveness and simple wet-lab protocol, although some criticism of this approach remains. Here we validated a protocol for pooled whole-genome re-sequencing (Pool-seq) of *Arabidopsis lyrata* libraries prepared with low amounts of DNA (1.6 ng per individual). The validation was based on comparing single nucleotide polymorphism (SNP) frequencies obtained by pooling with those obtained by individual-based Genotyping By Sequencing (GBS). Furthermore, we investigated the effect of sample number, sequencing depth per individual and variant caller on population SNP frequency estimates. For Pool-seq data, we compared frequency estimates from two SNP callers, VarScan and Snape; the former employs a frequentist SNP calling approach while the latter uses a Bayesian approach. Results revealed concordance correlation coefficients well above 0.8, confirming that Pool-seq is a valid method for acquiring population-level SNP frequency data. Higher accuracy was achieved by pooling more samples (25 compared to 14) and working with higher sequencing depth (4.1× per individual compared to 1.4× per individual), which increased the concordance correlation coefficient to 0.955. The Bayesian-based SNP caller produced somewhat higher concordance correlation coefficients, particularly at low sequencing depth. We recommend pooling at least 25 individuals combined with sequencing at a depth of 100× to produce satisfactory frequency estimates for common SNPs (minor allele frequency above 0.05).

## Introduction

The method of pooling biological samples for downstream analysis has been used for more than seventy years [1]. The main advantage of pooling is that more samples can be analyzed in a cost-effective way. Pooling has been widely used in population genetics analysis for the estimation of single-nucleotide polymorphism (SNP) frequencies (reviewed in Sham et al. [2]). More recently, the field of population genetics has been revolutionized by the development of next-generation sequencing (NGS), as it is now possible to study genetic variation at the whole-genome level [3–7]. Whole-genome sequencing of pooled DNA is more recent and known as Pool-seq [8]. While this method has become popular in the last few years, it has also been questioned, particularly in regard to the accuracy of SNP frequency data it produces [9,10]. To address this criticism, we investigated the robustness of Pool-seq in estimating SNP frequencies depending on sample size, sequencing depth and the SNP caller used.

So far, Pool-seq has been used in the study of bacteria [11], yeast [12], flatworm [13], sea urchins [14], plants [15,16], *Drosophila* [17–19], fish [20], birds [21] and mammals [22–25]. The approach has been applied to identify genomic loci affecting a trait of interest [19], to infer the demographic history of populations [20], to detect the signature of selection [17,18,25] and to perform genome-wide association studies (GWAS) [15,16]. In many cases, the pooling of samples is used to reduce costs. But pooling can be obligatory in other cases, such as when separating individuals is problematic [14,26] or when there is insufficient DNA to make individual libraries.

Several weaknesses of the method have been discussed. Low individual numbers, rough DNA quantification, and low sequencing depth can add error to polymorphism frequency estimates [27,28]. While these problems can be resolved and/or the magnitude of impact estimated, there are two more systemic, less easily resolvable limitations. When DNA of individual samples is pooled, information on haplotypes is lost. It is no longer possible to link a polymorphism with the individual to which it belongs [8], which is a problem for studies that require information on linkage disequilibrium, for example. The other limitation is that sequencing errors cannot easily be distinguished from true rare alleles [9]. Several authors have developed statistical approaches to tackle these two issues [29–33], which have been implemented in software programs to analyse pooled data [34–37]. In line with the intention of such improvements, the goal must be to assess the impact of problems of Pool-seq and to come up with procedures to resolve them, especially as whole-genome re-sequencing of individuals for population genomics is still expensive for species with medium-sized to large genomes.

This study focused on validating Pool-seq for population genomics by comparing SNP frequencies revealed by pooling and re-sequencing with those revealed by individual-based Genotyping By Sequencing (GBS) [38]. Comparisons were based on field-sampled plants of *Arabidopsis lyrata*. Library preparation required very little DNA and was performed with standard laboratory equipment. The three main questions we addressed were: (1) What is the increase in accuracy of Pool-seq SNP frequency estimates when increasing pool size? (2) What is the sequencing depth per individual required to obtain reliable population SNP frequencies with Pool-seq? And, (3) what is the difference in accuracy of SNP calling between a heuristic approach as implemented in the software VarScan [39] and a Bayesian approach as implemented in Snape [35]?

## Materials and Methods

**Fig 1:**
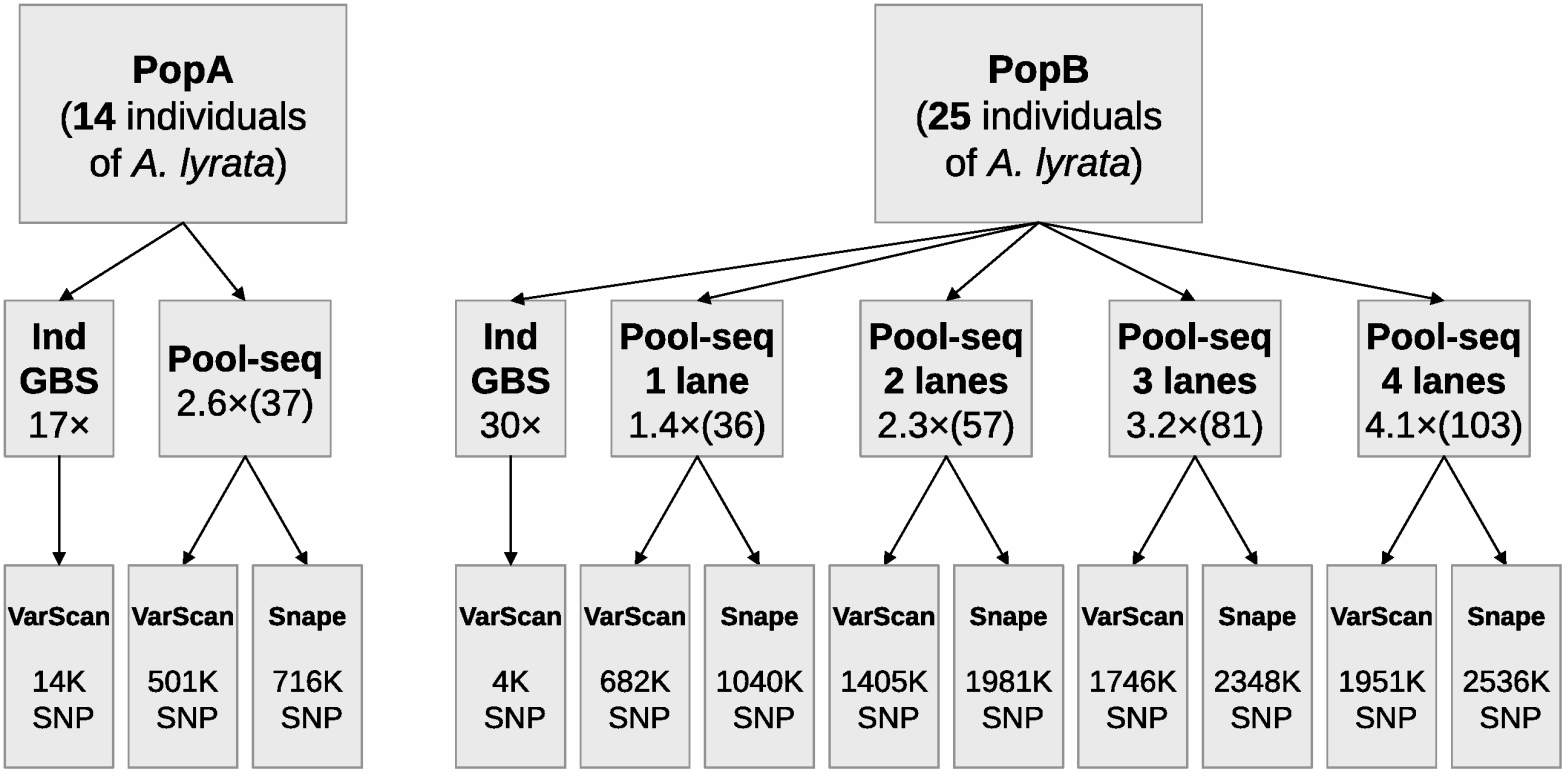
Diagram presenting the data sets produced to validate pooled whole-genome re-sequencing (Pool-seq) by individual-based Genotyping By Sequencing (GBS). The three rows of boxes contain the following information: top row: name of *Arabidopsis lyrata* population and number of individuals per population; second row: sequencing method, number of lanes merged (Pool-seq, population B only), the sequencing depth per individual and per pool (in parentheses); third row: the number of SNPs called by VarScan and Snape for each data set. Note that for GBS data, only the SNP caller VarScan was used.

The *A*. *lyrata* plants of population A were collected in Presque Isle State Park (Erie, PA, USA) with a permit granted by the Commonwealth of Pennsylvania. The *A*. *lyrata* plants of population B were collected in the Clark Reservation State Park (Jamesville, NY, USA) with a permit granted by the New York State Office of Parks, Recreation and Historic Preservation. DNA of field-collected plants was extracted from silica-dried leaves with the DNeasy 96 Plant Kit (Qiagen, Hombrechtikon, Switzerland). Each DNA sample was quantified twice with the DNA quantification kit Quant-IT^TM^ DNA HS (Invitrogen, Paisley, UK), a method based on fluorimetry with a DNA-specific dye. Samples were only accepted as suitable for the study if the average concentration was at least 0.25 ng/ml and the coefficient of variation between the two rounds of quantification was smaller than 0.1. We sampled 14 individuals from population A and 25 from population B (Fig 1). The same individuals of these two populations were analysed by pooled (Pool-seq) and individual (GBS) sequencing.

### Library preparation: Pool-seq

Libraries for Pool-seq were prepared with the Nextera Kit (Illumina, San Diego, CA, USA) from equimolar-pooled DNA samples for each population. For each library a total of 40 ng of DNA was used, 2.8 ng per individual for population A and 1.6 ng per individual for population B. The protocol was customized to work with strips of 8 PCR tubes. The tagmentation time was increased from the manufacturer’s protocol of 5 min to 10 min. The number of PCR cycles was increased to 8 (instead of 5) and the elongation time was decreased to 2 min (instead of 3 min). Library A was paired-end sequenced for 100 bases (PE100) on half a lane of Illumina HiSeq2000. Library B was PE100 sequenced on four lanes, each time constituting one quarter of the lane. Data of the lanes of population B were merged to create combinations from one to four lanes together (lane 1, lanes 1+2, lanes 1+2+3, lanes 1+2+3+4; Fig 1).

### Library preparation: GBS

Genomic DNA (50 ng per individual) was digested at 37°C for 65 min in a 20 μL reaction volume with 5 U *Msp*I (NEB, Ipswich, MA, USA) in 10× NEBuffer 4. Following heat inactivation of the restriction enzyme (65°C, 20 min), tubes were allowed to cool slowly to room temperature covered with tinfoil. Adapter ligation was then performed immediately, using the following reaction mixture: 5 μL 10× NEBuffer 2, 1.93 μL P1 adapter (10 μM; sequence as per Elshire et al. [39] but with a CG instead of a CWG sticky end, and containing a 4–9 base barcode sequence), 1.93 μL P2 adapter (10 μM; sequence as per Elshire et al. [38] but with a CG instead of CWG sticky end), 1.8 μL rATP (100 mM), 1.5 μL T4 DNA ligase (2×10^6^ U/mL), made up to 50 μL with ddH_2_O. Ligation reactions were incubated at room temperature for 45 min, then heat-inactivated at 65°C for 20 min. Tubes were allowed to cool slowly as before.

To multiplex barcoded samples, 5 μL of each ligation mix was pooled. The mixture was cleaned with a Clean and Concentrator -5 Kit (Zymo Research, Irvine, CA, USA), eluted in 50 μL Buffer EB. The pooled and cleaned DNA was used as template in 25 parallel PCR amplifications (replicated to minimise template bias). Each well included 2 μL template DNA, 2.5 μL of each PCR primer (as per Elshire et al. [38]), 5 μL dNTPs (2 mM), 0.5 μL Taq polymerase (Promega, Madison, WI, USA), 5x GoTaq buffer (Promega) and ddH_2_O to a final volume of 50 μL. Cycling protocol was as follows: 72°C for 5 min, 96°C for 30 s, 18 cycles of [96°C for 30 s, 65°C for 30 s, 72°C for 30 s], and a final extension of 72°C for 5 min. All replicate PCR reactions were pooled, and cleaned a second time as before, eluting in 30 μL of buffer per ~200 μL of PCR product. Size selection was performed with the Caliper LapChip XT (PerkinElmer, Waltham, MA, USA), set to collect two peaks (first peak: 350 bp, second peak: 455 bp), which effectively collected fragments between 301–519 bp due to the machine’s size accuracy limit of 14%. A third cleanup was performed, eluting in 17 μL Buffer EB. Sequencing was performed in a single Illumina HiSeq2000 lane.

### Bioinformatics pipelines and SNP frequency comparison

The bioinformatics pipelines for Pool-seq and GBS sequence data were kept as similar as possible to minimize differences due to software used (pipelines accessible at: http://github.com/fraca). The sequences are stored at the European Nucleotide Archive (http://www.ebi.ac.uk/ena) with the accession number PRJEB8335. Demultiplexing of the GBS data was performed with the preprocess_radtags script of Stacks [40], which retains reads with the proper barcode and restriction cut sites.

The Pool-seq and GBS sequences were trimmed using the script trim-fastq.pl of the software program PoPoolation [34] with a base quality threshold of 20, trimmed only from the 3' end to allow the subsequent removal of duplicates. Reads were mapped with BWA-MEM using default parameters [41]. The first 8 scaffolds of the published genome of *A*. *lyrata* v1.0 [42] were used as the reference genome. Data of the Pool-seq lanes of population B were merged to create the different combinations. Duplicate reads were removed with the MarkDuplicates tool of Picard [43]. Only proper paired reads with a mapping quality score above 20 were retained to create a pileup file with SAMtools [44]. The pileup file of Pool-seq data was filtered to retain regions with depth of coverage per site of 14–500 for population A and 25–500 for population B. The pileup file of GBS data was filtered for regions with depth of coverage per site of 5–500 for an individual and for data available for at least 90% of the individuals of a population. The regions near insertions and deletions were identified (identify-genomic-indel-regions.pl) and removed (filter-pileup-by-gtf.pl) with PoPoolation [34]. The genomic interspersed repeats were identified in the reference genome with RepeatMasker [45] using the default settings for “arabidopsis” and removed from the pileup files.

Finally, the filtered pileup files were used to call SNPs with the program VarScan with a significance (*P*) value ≤ 0.05, minimum base quality of 20 and a minimum allele count of two reads. For the Pool-seq data, SNPs were additionally called with Snape [35]. We retained SNPs with a posterior probability of segregation > 0.9 and minimum allele count of two reads. The nucleotide diversity and the genetic differentiation from the reference genome that are needed to set prior probabilities in the Bayesian model of Snape were calculated by NPStat [37]. We used the BEDTools software [46] to calculate sequencing depth or depth of coverage per site, defined as the number of times each base was sequenced per individual or per population pool. We applied the same thresholds for SNP calling and genome coverage calculation. Fig 1 presents the final 12 data sets used for further analysis. Allele frequency estimates were calculated as the fraction of reads carrying the non-reference allele for Pool-seq data, and the fraction of the non-reference allele across GBS-derived genotypes.

Three statistics were used to compare Pool-seq-based SNP frequencies with those obtained by GBS. First, the concordance correlation coefficient (CCC) was calculated using the epiR package [47]. This test statistic can be used to evaluate the agreement between two variables [48]. The CCC combines precision (deviation from best-fit-line) and accuracy (deviation of best-fit-line from 45° line through origin) to determine how far the observed data deviate from the line of perfect concordance. Second, the absolute value of the difference between the estimated SNP frequencies with the two methods (|Δf|) was calculated and its distribution investigated. Third, a false negative rate was calculated as the fraction of SNPs called in GBS but not in the pooled sample, relative to the total number of SNPs called by GBS. This calculation included only genomic regions covered by both GBS and Pool-seq data, and considered SNP frequencies estimated from GBS to represent the true population frequencies. Because sequencing depth of GBS reads did not meet the minimum threshold of five reads for all the individuals, data did not allow the reliable estimation of a false positive rate of SNP calling.

## Results

### Sequencing statistics

Pooled sequencing of population A yielded 34 million paired-end reads. Prior to restricting the reads falling within an informative range of coverage depth, 50% of reads mapped unambiguously to 74% of the *A*. *lyrata* nuclear genome, at a mean depth of 27×. After applying the read depth cutoff (min 14×, max 500×) and removing duplicates, 46% of the reads mapped to 41% of the *A*. *lyrata* nuclear genome. The mean sequencing depth of population A was 37× in the final data set, which is equivalent to a mean depth of 2.6× per individual. Pooled sequencing of population B was performed on four lanes, each of which yielded ~40 million paired-end reads. We unambiguously mapped 59% of the total reads to cover 80% of the *A*. *lyrata* nuclear genome, at a mean depth of 25× per lane. After applying the read depth cutoff (min 25×, max 500×) and removing duplicates, the percentage of the genome covered by one lane was on average 36%, while the four lanes together covered 70%. The mean sequencing depth (post-cutoff) of population B depended on the number of lanes merged; depth was on average 36× for one lane and 103× for four lanes (Fig 1). Accordingly, sequencing depth per individual varied between 1.4× and 4.1×. Individual sequencing by GBS yielded 105 million paired-end reads (population A and B together) that were correctly barcoded and trimmed. We unambiguously mapped 40% of reads to cover 2% of the *A*. *lyrata* nuclear genome. Once the read depth cutoff (min 5×, max 500×) was applied, the mean sequencing depth per individual for population A in the final data set was 17× (range across individuals: 10×-30×). For population B the mean sequencing depth per individual was 30× (range across individuals: 11×-113×).

### Number of SNPs

**Fig 2:**
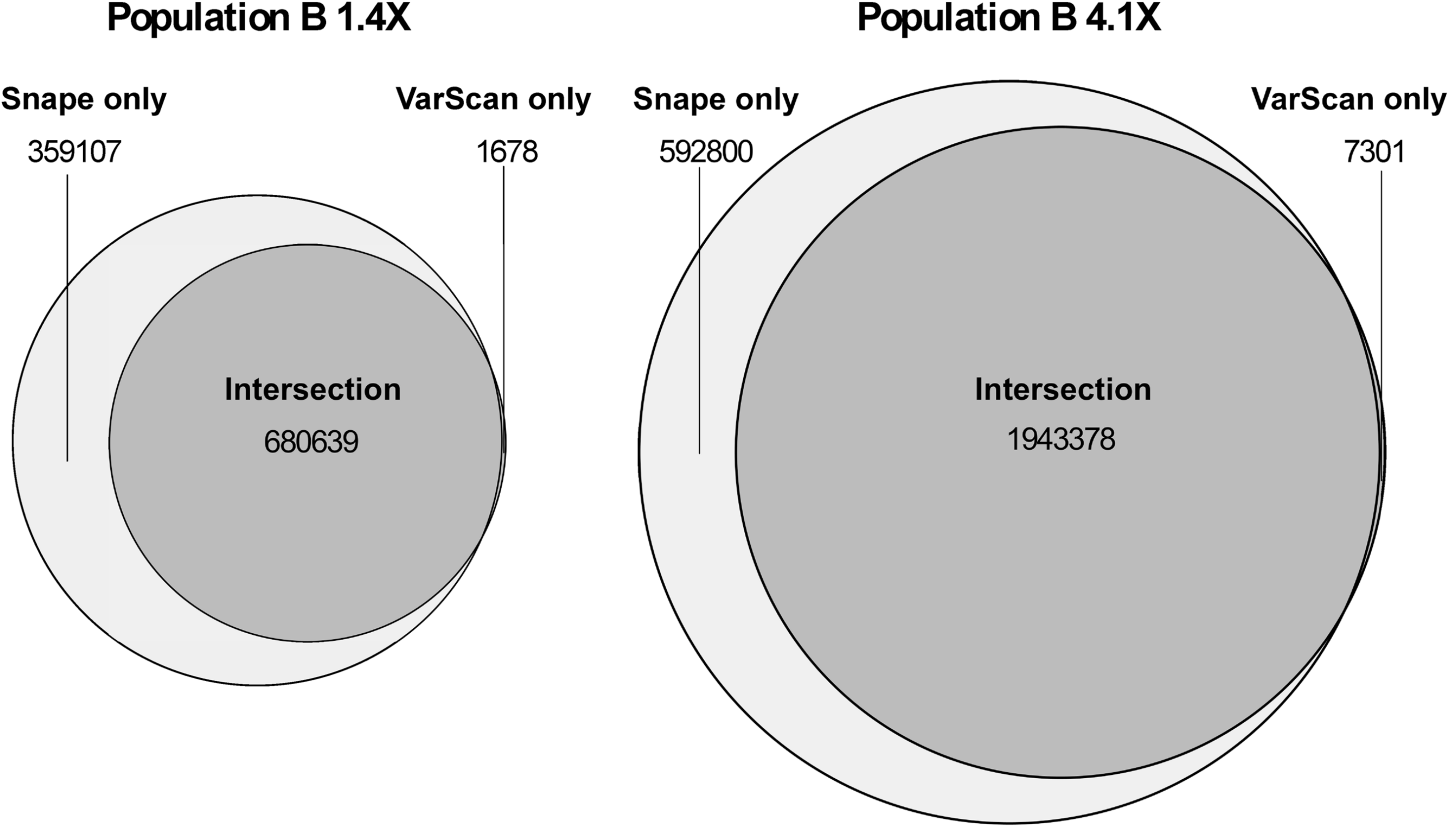
Venn diagram of Pool-seq SNPs called with VarScan (dark grey) and Snape (light grey). The left-hand panel shows the SNPs called for population B using data from lane 1 only. The right-hand panel shows the SNPs called for population B with the data from all four lanes. The figure was produced with the R package VennDiagram [49].

Table 1 shows the number of SNPs called by GBS and Pool-seq. For the Pool-seq protocol and population A, the software VarScan called 0.50 million SNPs, while Snape called 0.72 million SNPs. Increasing the depth from 1.4× to 4.1× (from one to four lanes) for population B increased the number of SNPs called. Using VarScan, the SNPs called increased from 0.68 million to 1.95 million. Using Snape, the SNPs called increased from 1.04 million to 2.54 million. Fig 2 shows the fraction of SNPs called with both VarScan and Snape in population B using one or four lanes. Almost all the SNPs called by VarScan were also called by Snape. The percentage of SNPs called by both programs relative to the total number of SNPs called by either Snape or VarScan, increased from 65% to 76% when the input data were increased from one lane to four lanes.

**Table 1:** Comparison of SNP numbers and frequency estimate accuracy revealed by Pool-seq and by GBS. Columns report: library/lane identity (population A or B, estimation of sequencing depth per individual in Pool-seq, and software used to detect SNPs of Pool-seq data set), number of SNPs detected by GBS (SNP_GBS_) and Pool-seq (SNP_Pool-seq_), overlapping number of SNPs detected (SNP_both_), concordance correlation coefficient (CCC) with lower and upper 95% confidence limit (LCL; UCL) of CCC, the mean of the absolute difference in SNP frequency estimates of the two methods (|Δf|), false negative rate (FN rate), that is, the fraction of SNPs called by GBS but not by Pool-seq, and their mean minor allele frequency (FN MAF).

Library/lane ID	SNP_Pool-seq_	SNP_GBS_	SNP_both_	CCC	LCL	UCL	|Δf|	FN rate	FN MAF
A 2.6× VarScan	500’515	13’843	5731	0.827	0.819	0.835	0.109	0.270	0.115
A 2.6× Snape	716’483	13’843	7102	0.864	0.858	0.870	0.103	0.137	0.075
B 1.4× VarScan	682’317	4177	1333	0.887	0.876	0.898	0.092	0.385	0.077
B 1.4× Snape	1’039’746	4177	1754	0.911	0.902	0.918	0.083	0.212	0.054
B 2.3× VarScan	1’405’122	4177	2166	0.931	0.926	0.937	0.073	0.287	0.059
B 2.3× Snape	1’981’376	4177	2636	0.941	0.937	0.946	0.067	0.146	0.043
B 3.2× VarScan	1’745’682	4177	2413	0.946	0.942	0.950	0.063	0.211	0.049
B 3.2× Snape	2’348’269	4177	2738	0.951	0.948	0.955	0.059	0.116	0.038
B 4.1× VarScan	1’950’679	4177	2536	0.952	0.948	0.955	0.058	0.170	0.045
B 4.1× Snape	2’536’178	4177	2771	0.955	0.952	0.958	0.055	0.101	0.036

GBS led to more SNPs for population A than for population B. The smaller sample of individuals in population A (14 instead of 25 in population B) made it easier to attain the processing threshold of five or more reads for at least 90% of individuals. Therefore, population A had higher overlap among individuals in genomic regions with sufficient sequencing depth and a higher total number of called SNPs. Moreover, the number of SNPs identified by both GBS and Pool-Seq was low (column SNP_both_, Table 1) because GBS revealed SNP information for a small fraction of the genome and that fraction overlapped incompletely with genomic regions also covered with acceptable depth by Pool-seq.

### Comparison of SNP frequencies revealed by Pool-seq versus GBS

**Fig 3:**
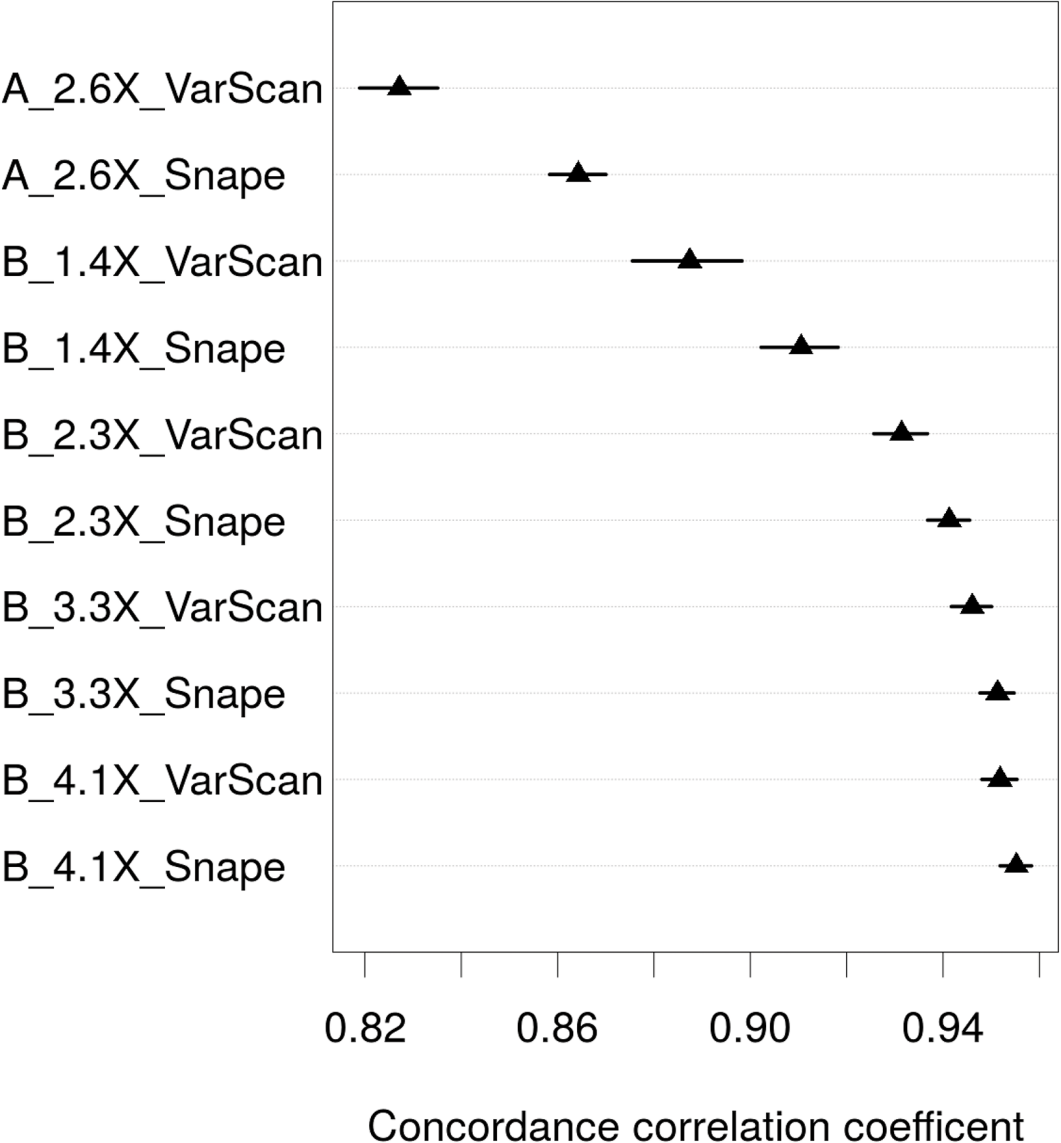
Concordance correlation coefficient between SNP frequencies estimated with Pool-seq and GBS for each library/lane combination and SNP caller. Mean CCC values with upper and lower 95% confidence ranges are shown. The name of a library/lane combination contains information on: the population (A or B), sequencing depth per individual by Pool-seq, and the software used to detect SNPs for Pool-seq (either VarScan or Snape; for GBS, only VarScan was used).

First, SNP frequencies obtained with Pool-seq and GBS were compared by the use of the concordance correlation coefficient (CCC), which captures the agreement between two variables by accounting for precision and accuracy and which can range from 0 to 1. Fig 3 illustrates CCC values with upper and lower 95% confidence ranges for all library/lane combinations studied. CCC values for population A were 0.827 for SNPs called with VarScan and 0.864 for those called with Snape (Table 1). For population B, CCC values increased with increasing depth of coverage per site from 0.887 (1.4×) to 0.952 (4.1×) with VarScan and from 0.911 (1.4×) to 0.955 (4.1×) with Snape. S1 Fig illustrates the correlation between SNP frequency estimates of Pool-seq and those of GBS. The correlation between the two increased when more samples were pooled, and when the depth of coverage per site was increased.

**Fig 4:**
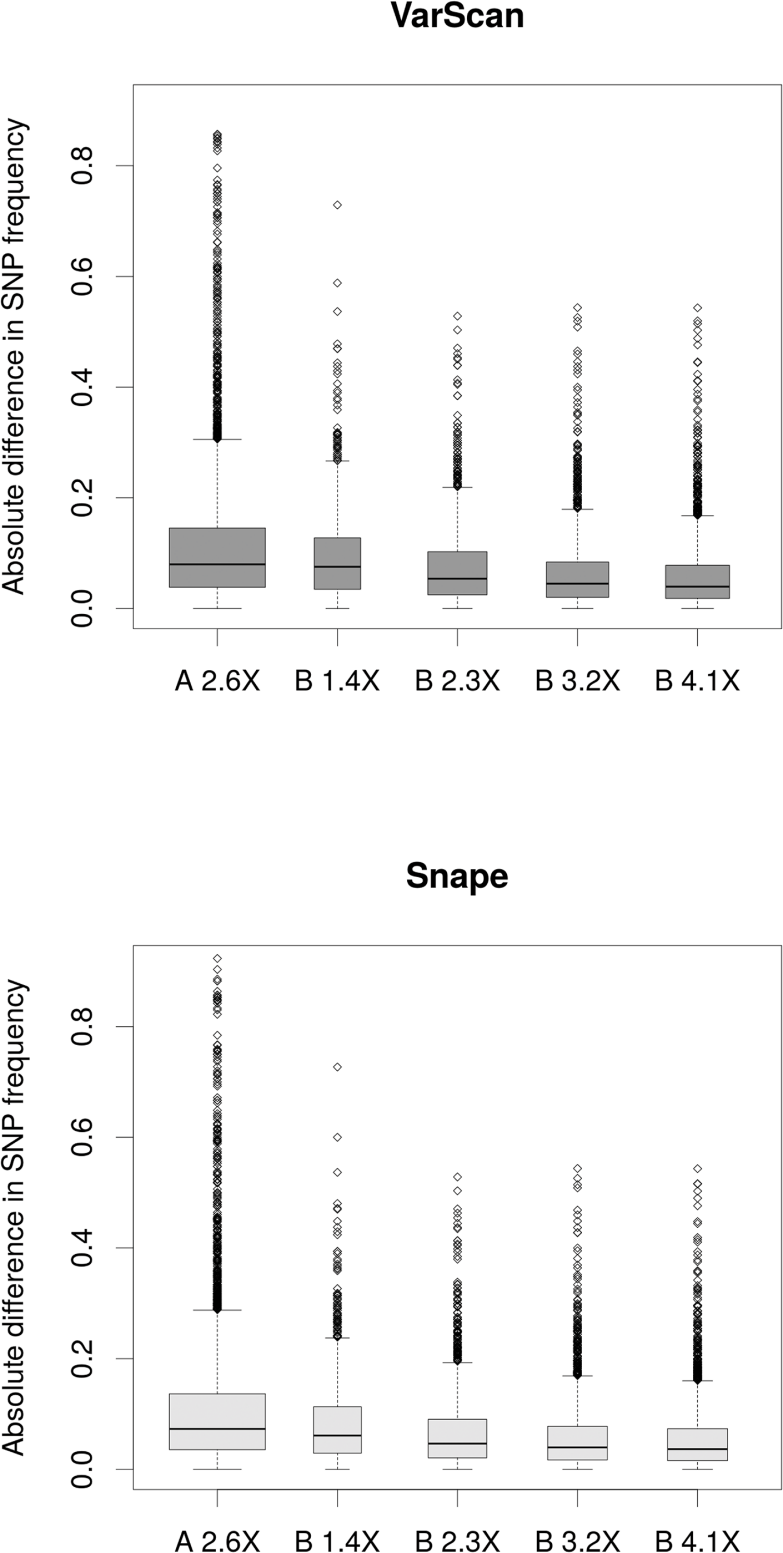
Box plot illustrating the distribution of the absolute difference in SNP frequency estimates between Pool-seq and GBS. The upper panel (dark grey) shows distributions when SNPs were called with VarScan for Pool-seq, the lower panel (light grey) shows distributions with Snape. Library names contain information on: the population (A or B), and the sequencing depth by Pool-seq. The band inside each box shows the median, while the lower and upper ends indicate the first and third quartile, respectively. The lower whisker is -1.5x the interquartile range from the first quartile, while the upper whisker is +1.5x the interquartile range from the third quartile. The diamonds represent outliers.

Second, SNP frequencies revealed with Pool-seq and GBS were compared based on the absolute difference between the SNP frequency estimates of the two methods (|Δf| in Table 1). The mean |Δf| for population A was 0.109 with VarScan and 0.103 with Snape. The mean |Δf| for population B decreased with increasing sequencing depth, from 0.092 to 0.058 with VarScan and from 0.083 to 0.055 with Snape. Fig 4 shows the distribution of |Δf| for each library/lane combination, and S2 Fig presents the distribution of the difference between the SNP frequency estimates of the two methods across the achieved read depth at SNP sites for each library/lane combination. The difference in SNP frequencies between methods was generally lower when read depth was high, both across and within library/lane combinations. Furthermore, the distribution of the difference was not appreciably biased towards either negative or positive values (S2 Fig).

**Fig 5:**
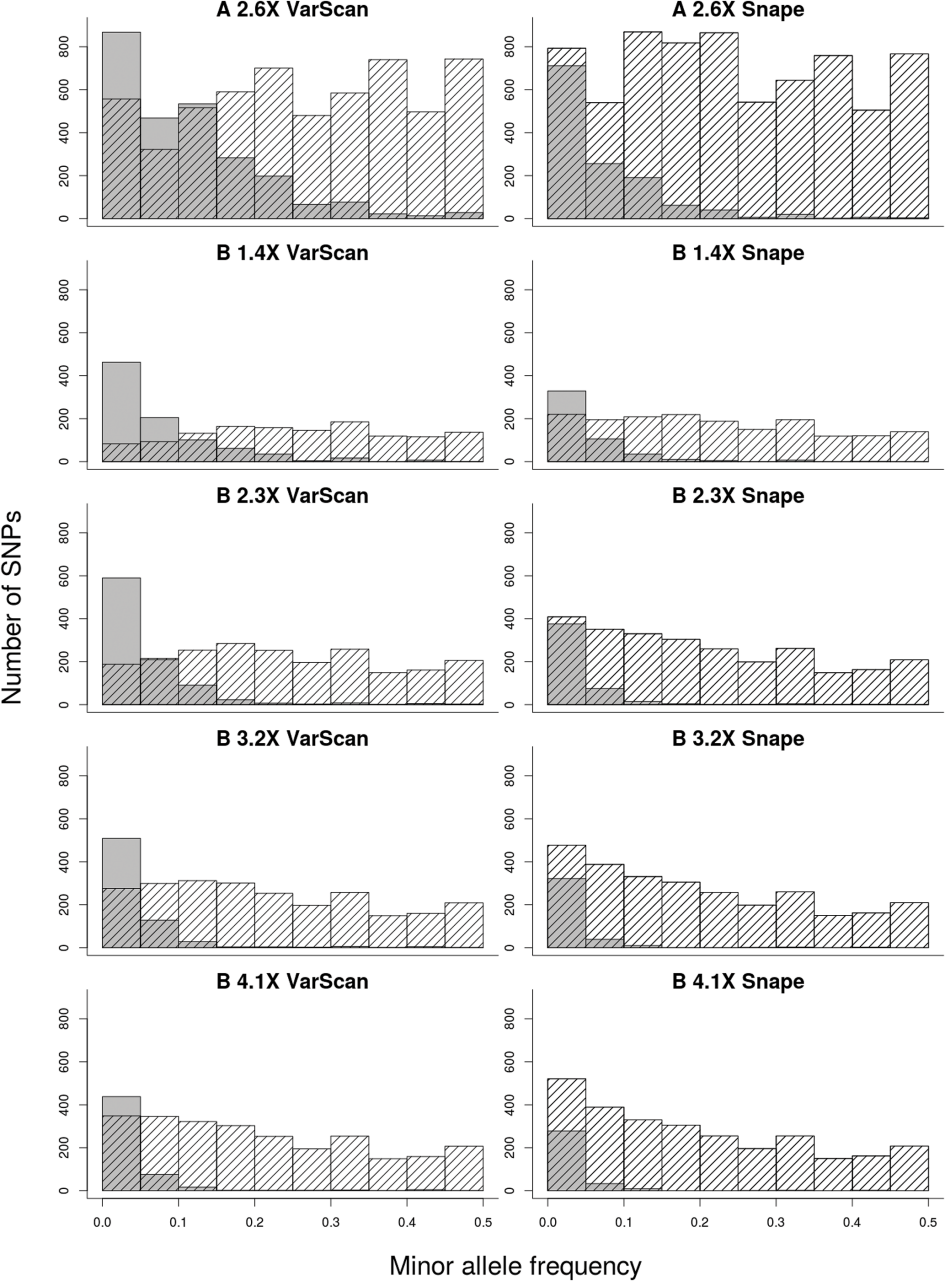
Histogram of minor allele frequency of GBS. The grey bars represent the SNPs present only in GBS. The striped bars represent the SNPs sequenced in the GBS and Pool-seq samples. The 10 panels show the results for the various Pool-seq library/lane combinations and the two SNP callers. The name of a library/lane combination contains information on: the population (A or B), sequencing depth per individual by Pool-seq, and the software used to detect SNPs for Pool-seq (either VarScan or Snape; for GBS, only VarScan was used).

Third, the false negative rate (FN rate in Table 1) decreased with increasing sequencing depth, from 0.385 (1.4×) to 0.170 (4.1×) with VarScan and from 0.212 (1.4×) to 0.101 (4.1×) with Snape. At the same time, the mean frequency of minor alleles at GBS SNPs that were missed by Pool-seq (FN MAF in Table 1) decreased from 0.077 to 0.045 with VarScan and from 0.054 to 0.036 with Snape. Fig 5 illustrates that the minor allele frequency at SNP sites missed by Pool-seq was mostly lower than 5% when the number of sequenced individuals and the sequencing depth per individual were both high.

## Discussion

Pooled whole-genome re-sequencing (Pool-seq) has only recently been adopted for population genomics in eukaryotes, so validation studies are needed, together with test of aspects of the wet-lab protocol and effects of the bioinformatics pipeline on results. Several studies have addressed the validation of this method (see Table 1 in [28]) but very few have examined the kind of large data sets now common in population genomics, containing more than a few thousand SNPs [22,27,32,51]. Here we analysed two populations of *Arabidopsis lyrata* by sequencing pools of individuals, and sequencing the same individuals separately by GBS. The main objective was to compare SNP frequencies obtained by Pool-seq with GBS-based SNP frequencies. Overall, we found that concordance correlation coefficients between SNP frequencies based on the two methods were high, between 0.827 and 0.955. These values are well within the range of other validation studies of pooled sequencing (e.g. Table 1 in [28]). Concordance increased with the pool size, with mean individual sequencing depth in the pool, and with the use of Snape as compared to VarScan as SNP calling software for the pooled samples.

The comparison of different numbers of individuals pooled was based on comparing 14 individuals with sequencing depth per individual of 2.6× and 25 individuals sequenced on two lanes with sequencing depth per individual of 2.3×. With the frequentist SNP caller VarScan, the concordance correlation coefficient increased from 0.827 to 0.931, while the mean absolute difference between SNP frequency estimates from the two methods decreased from 0.109 to 0.073 (Table 1, Figs 3 and 4). With the Bayesian-based SNP caller Snape, the concordance correlation coefficient increased from 0.864 to 0.941, while the mean absolute difference between SNP frequency estimates from the two methods decreased from 0.103 to 0.067. These results clearly show that an increase in the number of individuals that are pooled—at least for the range we worked with—improves the accuracy of SNP frequency estimation, as predicted by several theoretical studies [8,10,33]. Similar to our results, those of another study on pooling different numbers of isofemale lines of *Drosophil*a revealed increases in concordance correlation coefficients from 0.822–0.867 with 22 lines to 0.906–0.934 and 0.911–0.936 with 42 lines [27]. Aside from this, we found that increasing the number of pooled individuals did not greatly increase the chance of detecting SNPs, at least not with sequencing depth per individual used here. The false negative rate remained almost unchanged, increasing slightly from 0.270 to 0.287 with VarScan, and from 0.137 to 0.146 for Snape.

The comparison of varying depth of coverage per site revealed further potential for improving SNP frequency estimates. An increase of the depth of sequencing per individual from 1.4× to 2.3×, 3.2×, and 4.1×, led to an increase in concordance of Pool-seq with GBS (Fig 3) and a decrease in the absolute difference between SNP frequency estimates between methods (Fig 4) and false negative rate (Table 1). In line with our results, a sequencing study on a pool of 30 individuals of the pine processionary moth [32] revealed improved frequency estimates when the sequencing depth was increased from a range of 10×-50× to >200×, equivalent to a depth per individual of 0.3×-1.7× to >6.7×. The authors observed an increase in the correlation coefficient from 0.93 to >0.99 (across different sequencing depths per individual for individual sequencing) and a decrease of the median of the absolute difference between individual-based and pooled-based frequency estimates from 0.067 to 0.007.

A major issue with the Pool-seq technique is a lack of power to detect rare alleles [9,27,33], which is unimportant for some applications but important for others. For example, rare alleles may be important for explaining phenotypic variation within populations [52] and therefore desirable to detect in genome-wide association studies. We investigated this issue by analyzing the minor allele frequency of false negative SNPs (SNPs that were called only in GBS but not in the Pool-seq samples). In all library/lane combinations, the majority of false negative SNPs had low minor allele frequencies (Fig 5). At the sequencing depth of 4.1× per individual in the pool with 25 individuals the majority of GBS SNPs not detected by Pool-seq had a frequency below 0.05 (mean = 0.045 for VarScan and mean = 0.036 for Snape; Table 1). For higher GBS-based SNP frequencies, the number of SNPs missed by Pool-seq rapidly decreased. This result supports the utility of our upper pool size and maximum depth of sequencing. It has been suggested that to detect a minor allele with near-certainty, its frequency must be larger than 10 divided by the number of pooled diploid individuals [33], which in our study would have been 0.4 for the larger population. We appeared able to detect all minor alleles with frequency > 0.15 at the largest pool size and sequencing depth tested (Fig 5). The discrepancy is likely due to the difference in variant calling approaches and the fact that we used a *P* = 0.05 threshold for detection as opposed to the *P* = 0.001 level used by Lynch et al. [33]. For some population genetics studies this detection threshold is likely to be acceptable and our results confirm that this kind of pooled data is useful for detecting common minor alleles. Of course, those considering Pool-seq should be aware of the limitation of this approach in detecting rare alleles.

Several SNP callers can be applied to pooled data (reviewed in [8]). We used VarScan [38], which uses a frequentist approach, and Snape [35], which uses a Bayesian approach. Both take into account sequencing depth, base quality, and statistical significance, while Snape includes information on nucleotide diversity and divergence from the reference genome to detect SNPs. Our results show that Snape called considerably more SNPs than VarScan (Fig 2). The number of SNPs called by Snape that were confirmed by GBS was on average 20% higher than the number of SNPs called by VarScan confirmed by GBS (column SNP_both_ in Table 1). Furthermore the false negative rate was found to be systematically lower with Snape. Therefore, it can be argued that Snape is more powerful at detecting SNPs than is VarScan. This may however be accompanied by an increase in the false positive rate, which is an important avenue for further investigation. Also, the concordance correlation coefficients between GBS and Pool-seq SNP frequencies were slightly higher with Snape than with VarScan, although this difference between SNP callers declined with increasing sequencing depth (Table 1, Fig 3). The absolute difference in SNP frequencies between methods was lower with Snape than with VarScan. These results indicate that the use of priors for nucleotide diversity and divergence contribute positively to the calling of SNPs.

In conclusion, we have presented a method that uses low input DNA (1.6 ng per individual) and widely-available commercial kits to perform pooled whole-genome re-sequencing. Thanks to the tagmentation step, we avoided fragmentation by sonication, which requires more input DNA. We validated SNP frequencies by comparison with GBS data. Our study strengthens the conclusion that the quality of pooled sequencing data sets relies on two critical parameters: the number of individuals that are pooled, and sequencing effort. In a recent review on Pool-seq [8], the authors recommend pools of at least 40 individuals with sequencing depth of more than 50× per pool. Lynch et al. [33] used a maximum likelihood estimator and suggested more than 100 individuals and a sequencing depth of 100× per pool to obtain high confidence in allele frequency estimates. Based on the empirical comparison we performed, we find that a pool of 25 individuals combined with a sequencing depth of 100× produces SNP frequency data with satisfactory precision and accuracy. We confirm that Pool-seq is a useful method to detect genomic variants with a frequency of about 0.05 and larger.

## Supporting Information

S1 Fig Scatter plots of SNP frequency estimates based on GBS and Pool-seq for the various library/lane combinations and the two SNP callers. The name of a library/lane combination contains information on: the population (A or B), sequencing depth per individual by Pool-seq, and the software used to detect SNPs for Pool-seq (either VarScan or Snape; for GBS, only VarScan was used). The solid line indicates the expectation of equal frequency with both sequencing approaches.
(EPS)

S2 Fig Hexbin plots of the difference in SNP frequency estimates between Pool-seq and GBS with respect to the total read depth at SNP sites of Pool-seq. The name of a library/lane combination contains information on: the population (A or B), sequencing depth per individual by Pool-seq, and the software used to detect SNPs for Pool-seq (either VarScan or Snape; for GBS, only VarScan was used). Hexagons are shaded by SNP count according to the scale shown on the right. The figure was produced with the hexbin package in R [50].
(TIFF)
