# Supplementary figures and images for: Validation of Pooled Whole-Genome Re-Sequencing in Arabidopsis lyrata
Source: PLoS One. 2015 Oct 13;10(10):e0140462. doi: 10.1371/journal.pone.0140462 (PMC4604096; doi:10.1371/journal.pone.0140462)

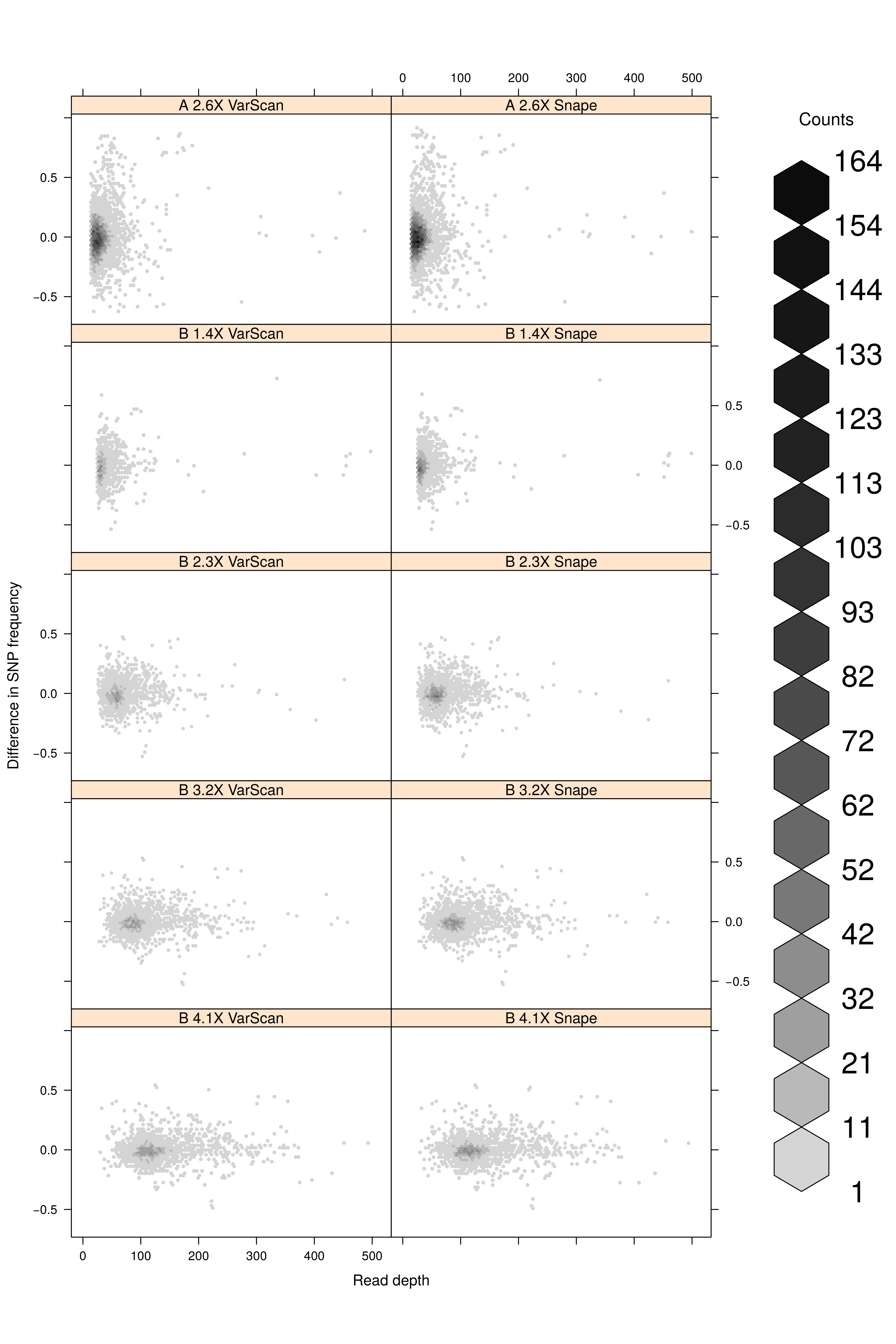

Supplement: S2 Fig — The name of a library/lane combination contains information on: the population (A or B), sequencing depth per individual by Pool-seq, and the software used to detect SNPs for Pool-seq (either VarScan or Snape; for GBS, only VarScan was used). Hexagons are shaded by SNP count according to the scale shown on the right. The figure was produced with the hexbin package in R [50]. (TIFF) [file pone.0140462.s002.tiff]
